# Enhanced Anti-Corrosion Performance of Co-Cr-Mo Alloy in Molten Al by Prior Oxidation Treatment

**DOI:** 10.3390/ma16237449

**Published:** 2023-11-30

**Authors:** Rongrong Shang, Biaobiao Yang, Yunping Li

**Affiliations:** 1 State Key Laboratory for Powder Metallurgy, Central South University, Changsha 410017, China; shangrr@csu.edu.cn; 2 IMDEA Materials Institute, C/Eric Kandel 2, Getafe, 28906 Madrid, Spain; 3 Department of Materials Science, Escuela Técnica Superior de Ingenieros de Caminos, Polytechnic University of Madrid, 28040 Madrid, Spain

**Keywords:** cobalt-based alloy, corrosion, molten Al, interfaces, oxide film

## Abstract

Co-based alloys are promising alternatives to replace the currently used tool steels in aluminum die-casting molds for producing sophisticated products. Although the reaction is significantly less severe compared to that of tool steels, bare Co-29Cr-6Mo (CCM) alloy is still gradually corroded under molten Al, leading to the local failure of the alloy due to the formation of intermetallic compounds between the matrix and molten Al. This study indicated that prior oxidation treatment at 750 °C on CCM alloy is beneficial in enhancing the corrosion resistance of the alloy to molten Al. The is more pronounced in the alloy after a longer oxidation treatment. However, after oxidation for longer than 24 h, the protectiveness of the film cannot be enhanced anymore. In addition, even after the full failure of the oxide film, the thickness loss rate of a sample with prior oxidation treatment is much lower than that of a bare sample. This can be attributed to the fact that network-aligned oxide particles of the cone structure boundary inhibit both the outwards movements of alloying elements and the dissolution of the intermetallic layer.

## 1. Introduction

Die casting is a crucial technique for producing high-accuracy and high-precision aluminum alloy components with excellent mechanical properties and a first-class surface finish. Hot-working tool steels are extensively used as die materials, but the reaction between the die and molten Al generally leads to the early failure of the die [1,2]. Due to their superior corrosion resistance to molten Al [1,3] and their exceptional mechanical properties at higher temperatures [4,5,6,7,8], Co-based alloys are promising alternatives to replace the currently used tool steels as aluminum die-casting molds for producing sophisticated products.

Although the reaction is significantly less severe compared to that of tool steels, bare Co-29Cr-6Mo (Co-Cr-Mo) alloy is still gradually corroded under molten Al, leading to the local failure of the alloy due to the formation of intermetallic compounds between the matrix and molten Al [9,10,11,12,13]. In light of this, various methods—including nitriding [14], oxidation treatment [10], and alloying Si [9,15], Al, Ti, and Zr [16]—have been adopted to improve the corrosion resistance of Co-Cr-Mo alloy to molten Al. Among these methods, oxidation treatment is the most economical, simple, and effective. After immersion in molten Al for 1 h, the thickness of an untreated Co-Cr-Mo alloy sample decreased pronouncedly from 2 to 1.345 mm, while no obvious thickness change was observed for an oxidation-treated one [10]. Tang et al. [10] attributed this corrosion resistance improvement to a compact and chemically inert oxide film on the sample surface that effectively protected the alloy from direct contact with molten Al for 2 h. Their work also revealed that after a long time of immersion, a cone-shaped structure—where the cone boundary evolved from the fragmented oxide film—will form and attenuate the protectiveness of the oxide film in molten Al [10]. 

In recent decades, various techniques such as X-ray photoelectron spectroscopy (XPS) have been used to characterize the oxide films on both Co-based alloys and Fe-based alloys [12,13,17,18,19,20,21,22,23,24]. In contrast to the single-layered oxide film in Fe-29Cr-6Mo alloy [17], a duplex oxide layer consisting of an outer CoO-rich layer and an inner Cr_2_O_3_-rich layer was observed in Co-Cr-Mo alloy subjected to oxidation at 750 °C for 4 h. With prolonged oxidation, CoO was gradually replaced by Cr_2_O_3_, resulting in a single-layered oxide film dominantly composed of Cr_2_O_3_ [13]. Tunthawiroon et al. [5] characterized the oxide films on Co-Cr-Mo-xSi alloys exposed to high-temperature oxidation, and the results indicated the key role of Si in stabilizing the Cr_2_O_3_-dominated film. In addition, the formation of CoO in the oxide film was inhibited obviously due to the enhanced selective oxidation of Cr by Si. Furthermore, Yang et al. [18] reported that the reaction interfacial structure between CoCrWAl_x_ alloy and molten Al was composed of five sublayers: alloy substrate, two product sublayers, a diffusion layer, and solidified Al, and the reaction products were identified as Al_9_Co_2_, Al_5_Co_2_, and Al_45_Cr_7_ phases. The variation in both microstructure and composition of the oxide film as a function of oxidation conditions clearly indicated the difference in corrosion resistance of the oxidation-treated alloys to molten Al.

Nevertheless, to the best of the authors’ knowledge, the influence of oxidation treatment duration on the interfacial reaction between Co-Cr-Mo alloy and molten Al is still unfortunately unascertained, although this point is very relevant to optimize the oxidation treatment parameters to design Co-Cr-Mo alloy that is highly corrosion-resistant to molten Al.

To fill this gap, under molten Al, the corrosion resistance of Co-Cr-Mo alloy after various oxidation treatments (in air at 750 °C for 0, 4, 12, 24, and 36 h) as well as the interfacial reaction between Co-Cr-Mo alloy and molten Al were systematically investigated in this work. A correlation between prior oxidation products and corrosion performance was established. This information provides a detailed picture of the evolution of the corrosion behavior of Co-Cr-Mo alloy in molten Al with various prior oxidation treatments.

## 2. Experimental

The material used in this study is composed of 29 wt.% Cr and 6 wt.% Mo, with the rest of the alloy mainly consisting of Co. Prior to oxidation, samples with dimensions of 20 × 5 × 2 mm^3^ were cut from a homogenized ingot (homogenization treatment: 1200 °C for 12 h) by electrical discharge machining, ground with SiC abrasive paper, and polished with a suspension of 0.3 µm alumina particles. Oxidation was carried out in air at 750 °C for 0, 4, 12, 24, and 36 h, using a muffle furnace. 

The prepared samples were immersed individually in molten Al at a temperature of 740 °C. After a static immersion for a given duration, the samples were removed and cooled in air. To quantitatively analyze the interfacial reaction, the thickness of the sample was measured 6 to 10 times in cross sections. Then, the thickness loss of the sample (Δ*X*) was calculated using the following equation:

Δ*X* = (*X*_0_ − *X*)/2 (1)

where *X*_0_ and *X* denote the mean thickness of sample before and after the immersion test, respectively. 

**Figure 1:**
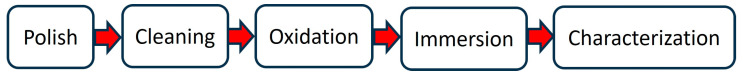
Experimental procedure of this study.

Microstructural observations and phase analysis were conducted using a field-emission scanning electron microscope (FE-SEM; S-3400N, Hitachi, Tokyo, Japan) equipped with an energy-dispersive spectroscope (EDS) and X-ray diffraction (XRD; X’Pert MPD, PANalytical, Almelo, The Netherlands) respectively. Cu Kɑ radiation with a wavelength of 0.1547 nm was used for XRD, and each sample was scanned from 20° to 70° with a scan rate of 1° per minute. For the FE-SEM measurement, the applied current and voltage were set at 45 kV and 40 mA, respectively. In addition, transmission electron microscopy (TEM; JEOL JEM-2100F, Hitachi, Tokyo, Japan) observation was performed using an ion slicer (JEOL EM-09100IS, Hitachi, Tokyo, Japan) from the section perpendicular to the reaction interface at 200 kV. Selected area diffraction patterns (SADP) were obtained for different phases and analyzed with the Crystallography 3.1 software. A schematic figure showing the research flow of this study is given in Figure 1.

## 3. Results

**Figure 2:**
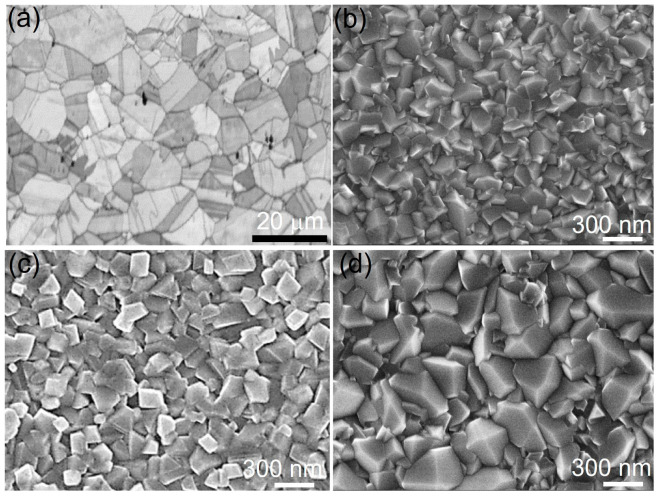
Surface morphologies of Co-Cr-Mo alloy after oxidation for (**a**) 0 h, (**b**) 4 h, (**c**) 12 h, and (**d**) 36 h, respectively.

Figure 2 depicts the surface morphologies of samples after oxidation for 0 h, 4 h, 16 h, and 36 h under FE-SEM. For the alloy without oxidation treatment, the sample surfaces are bright and smooth, and clear grain boundaries can be observed, suggesting no oxidation occurred on the sample surface (Figure 2a). After oxidation, all sample surfaces become dark under the naked eye and are characterized by angular oxide crystals stacking close to each other (Figure 2b–d). After oxidation for 4 h, the size of oxides ranges from 100–250 nm, while in the sample after oxidation for 12 to 36 h, all oxide sizes are comparable in a range of 250–500 nm, indicating the drastic growth of oxide crystals with prolonged oxidation. However, the growth in grain size of oxides after oxidation for longer than 12 h seems not as significant as that in the beginning. Because the oxide film profile at 36 h is comparable to that at 24 h, only the sample at 36 h is presented here.

**Figure 3:**
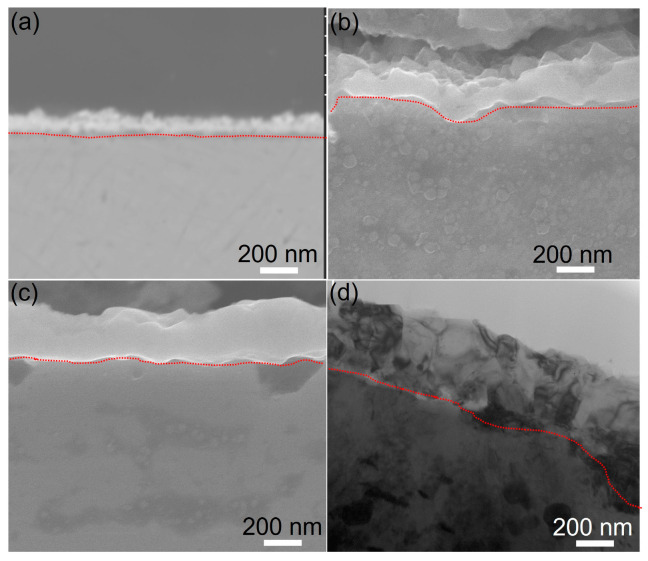
Cross-sectional FE-SEM morphologies of oxide films after oxidation for (**a**) 4 h, (**b**) 12 h, and (**c**) 36 h and (**d**) TEM bright image of the oxide film after oxidation for 36 h.

The cross sections of samples oxidized at 750 °C for various durations are shown in Figure 3, where the dashed lines indicate the oxide/matrix interface. The oxide film of the 4 h-treated sample exhibits a mean thickness of approximately 180 nm. The film seems not compact, as a large number of pores and cracks in both the surface and the cross section of the film are observed under FE-SEM. Films after oxidation for both 12 h and 36 h become thicker and more compact compared to the 4 h-treated sample. Both films demonstrate comparable thicknesses of about 350 nm. However, compared to the sample oxidized for 12 h, the film at 36 h is more compact in its cross section and smoother at the surface, suggesting that longer oxidation is beneficial to forming a defect-free and compact oxide film. The more detailed information of the film at 36 h is given in the TEM bright image of Figure 3d, suggesting that the film is very compact, and no obvious defect (crack or pore) can be observed in the interface between the oxide film and alloy matrix.

**Figure 4:**
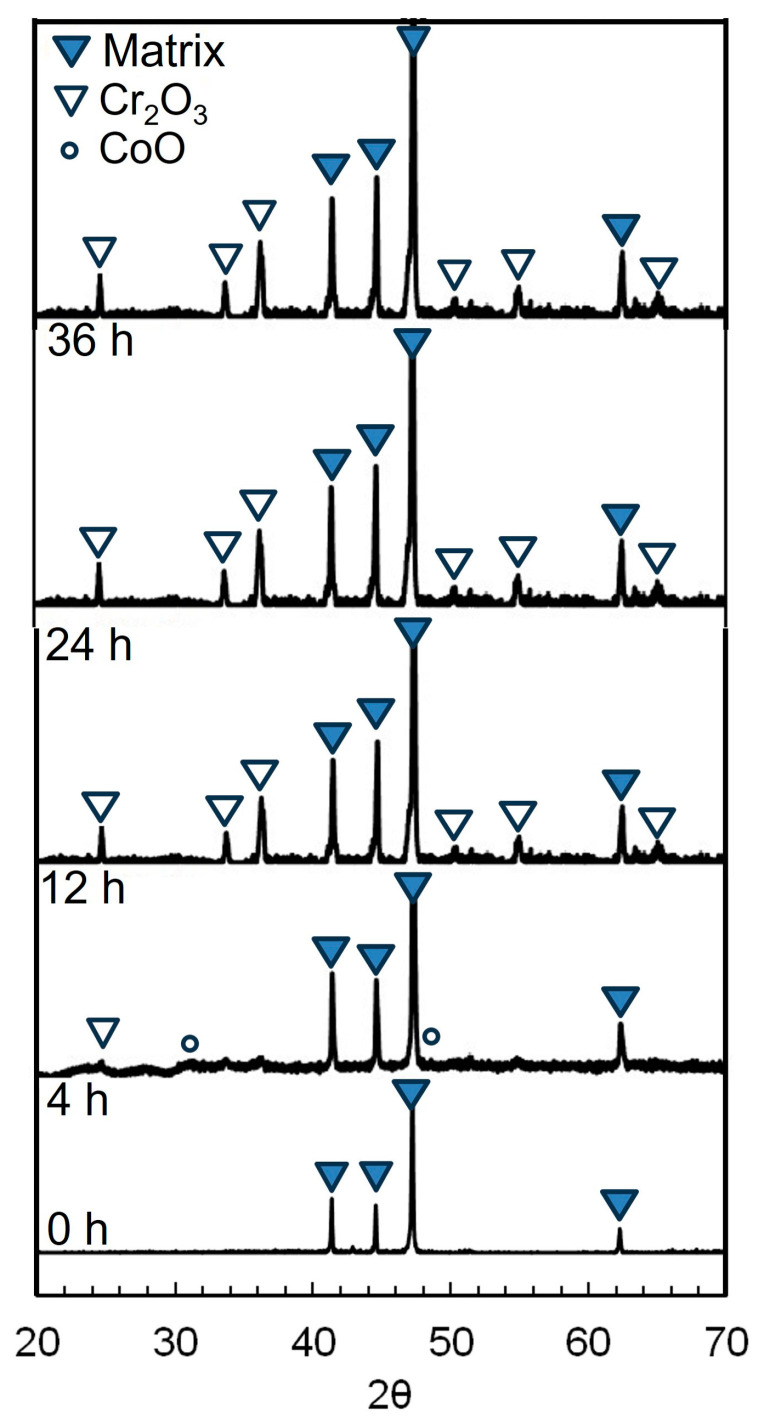
XRD patterns of Co-Cr-Mo alloys after oxidation for various durations.

The corresponding XRD results of the alloy after oxidation for various durations are presented in Figure 4. For the alloy before oxidation, only the peaks of the alloy matrix (ɛ-phase and γ-phase) are observed, indicating that no or only an extremely thin oxide film exists on the sample surface. For the sample oxidized for a short duration (4 h), apart from the dominant Cr_2_O_3_ phase, a few CoO phases were observed, which is in good agreement with the results obtained by using XPS [13]. After oxidation for longer than 4 h, the analysis revealed a dominant Cr_2_O_3_ phase in samples oxidized for 12 to 36 h. In addition, with prolonged oxidation, the peaks of the alloy matrix become weaker, suggesting the gradual thickening of the film. However, after oxidation for longer than 12 h, the relative peak strength of the film seemingly did not change significantly, suggesting the film thickness remains almost constant, which is consistent with the cross-sectional morphology of the film in Figure 3.

**Figure 5:**
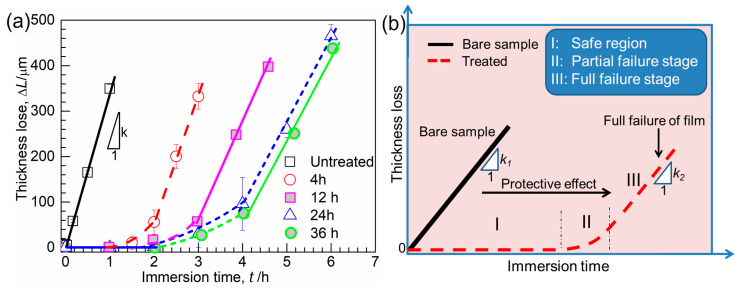
(**a**) Thickness losses of Co-Cr-Mo alloys after various oxidation treatments as a function of immersion time; for a comparison, the thickness losses of Co-Cr-Mo alloy without treatment are also plotted in the figure. (**b**) Schematic diagram showing the influence of oxidation treatment on corrosion resistance of alloy to molten Al.

After oxidation for 24 h the sample surface becomes darker and is homogenously covered by a compact oxide film. The non-treated sample surface after immersion is characterized by obvious geometrical variation. In addition, thick reactants are closely attached onto the sample surface, suggesting a severe reaction between the alloy matrix and molten Al. For the sample with prior oxidation, the sample geometry remains almost unchanged. This is especially obvious for the sample oxidized for a longer time, suggesting enhanced corrosion resistance of the alloy to molten Al. However, for the sample subjected to a short oxidation treatment, the immersed sample is characterized by a non-homogenous Al covering, in contrast to the sample with longer oxidation, implying a higher corrosion resistance of the alloy after a longer oxidation period. Nevertheless, even for the sample with prior oxidation for 36 h, obvious shrinking in the sample size and severe reaction between Al and the alloy matrix are observed obviously after immersion for 5 h. In summary, the longer the prior oxidation treatment, the higher the corrosion resistance of the alloy. Thickness losses of treated samples calculated from Equation (1) versus immersion time are shown in Figure 5. The thickness loss of the untreated sample approximately follows a linear law with immersion time, which is consistent to the results reported by Tang et al. [11,12] that the thickness loss of bare Co-Cr-Mo alloy is mainly controlled by the dissolution of the (Co, Cr, Mo)_2_Al_9_ intermetallic layer into molten Al. In contrast, the thicknesses of treated samples remained constant for a given period. With the progress of immersion, the film was destroyed locally and completely lost its protective effect finally by the attack of molten Al. 

In contrast to the linear thickness loss behavior of the untreated sample during immersion, the thickness loss–immersion time curve for treated samples was schematically divided into three stages as shown in Figure 5b: stage I, effective protection of the film due to no thickness loss of the sample; stage II, partial failure in the film with local thickness loss; as well as stage III, full failure of the film with thickness loss throughout the sample surface. With an increase in oxidation duration from 4 to 36 h, both periods of stage I and stage II were observed to be greatly increased, suggesting an improved protective effect of the oxidation film with prolonged oxidation. During stage III, the thickness loss of each sample follows a linear relationship with the immersion time, attributed to the direct reaction of the sample matrix with molten Al. When the oxidation duration is extended, stage III is gradually postponed (Figure 5), implying that the oxidation film accordingly has a better protective effect against corrosion. 

**Figure 6:**
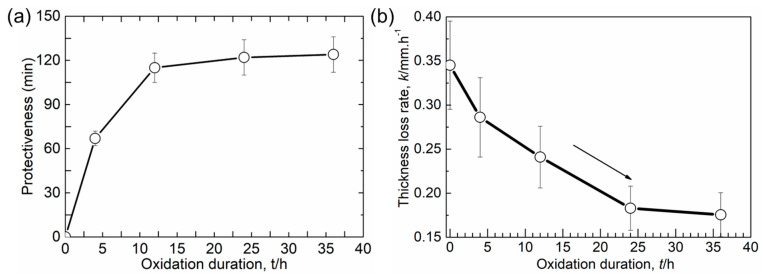
(**a**) Protectiveness of oxide film and (**b**) thickness loss rate of Co-Cr-Mo alloy matrix in stage III during immersion test as a function of oxidation duration.

The protectiveness (stage I) of the oxide film as a function of the oxidation time is plotted in Figure 6a. Oxidation treatment shows a significant influence in protecting the alloy matrix from corrosion, which is more pronounced in the sample after oxidation for longer than 12 h. However, this protective effect of the oxide film seems follow a parabolic behavior in relation to the oxidation time and reaches its maximum value after oxidation for 24 h. The thickness loss rates in stage III (slope of the line, *k*, as shown in Figure 5b) for samples were calculated as shown in Figure 6b as a function of oxidation time. The results demonstrate a decreasing trend with increasing oxidation duration. For instance, compared to the untreated sample, the thickness loss rate of the 24 h-treated sample is about half of that of the bare sample, suggesting a significant influence of the oxide film on corrosion even after its full failure. However, this influence seems to be greatly weakened in the sample after oxidation for longer than 24 h. The results in both Figure 6a,b suggest that further increasing the oxidation time of a sample could not effectively enhance the corrosion resistance of the alloy to molten Al. 

**Figure 7:**
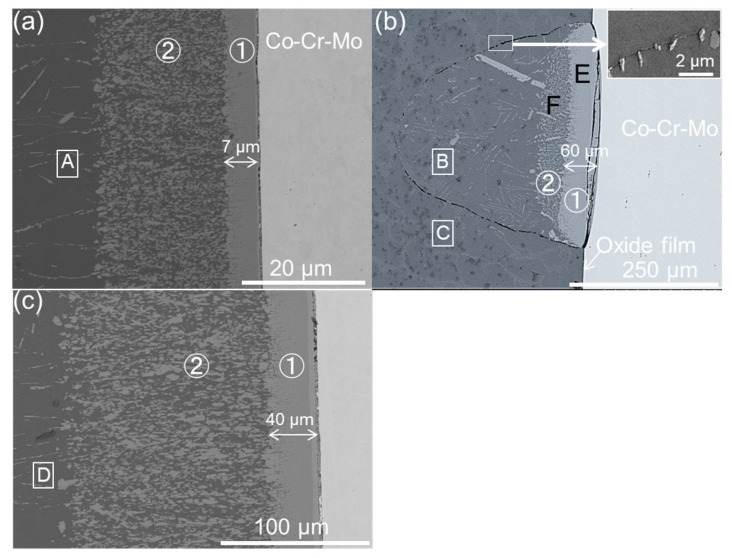
The interface structure of (**a**) untreated sample after immersion for 3 min, (**b**) 24 h-treated sample after immersion for 3 h (stage II in Figure 6b) in which an amplified figure was inserted to show the network-aligned cone boundary by the oxide particles, and (**c**) 24 h-treated sample after immersion for 5 h (stage III in Figure 6b). Note that areas A–D and areas E–F were selected to perform subsequent EDS analysis and TEM analysis, respectively.

Figure 7a–c display the typical interfacial structures for the untreated sample immersed for 3 min and the 24 h-treated sample immersed 3 h (stage II) and 5 h (stage III), respectively. For the untreated sample, two layers of reactants were observed: an intermetallic layer ① of (Co, Cr, Mo)_2_Al_9_ attached closely to the alloy matrix, and a multiphase layer ② between the intermetallic layer and Al [11,12]. The intermetallic layer possessed a clear and smooth interface with the alloy matrix but an uneven interface with the multiphase layer. The multiphase layer was composed of Al solution (dark in the image) and isolated phases (grey in the image) with irregular shapes and various particle sizes from 1 µm to 5 µm. 

In contrast to the homogenous reaction between the untreated sample and Al (Figure 7a), the failure of the treated sample takes place more preferentially in the oxide film with weak areas (defects in film). As shown in Figure 7b, a cone-shaped structure during stage II has a network-aligned boundary formed from the fragmented oxide film. In the interior of the cone, the duplex layer structure, similar to the untreated sample, is due to the direct reaction between the alloy matrix and molten Al. With the progress of immersion into stage III, the duplex layer structure spread to the whole interface (Figure 7c), indicating the film completely lost its protective effect finally due to the corrosion of molten Al. 

**Figure 8:**
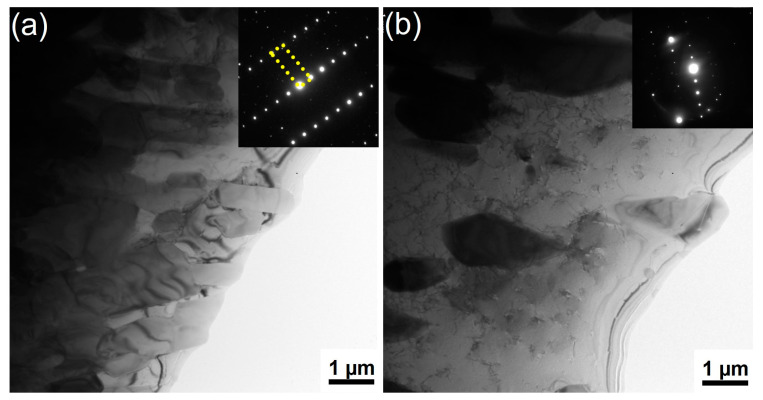
The TEM interface structure in the 24 h-treated sample after immersion for 3 h (**a**) the TEM image in the intermetallic layer (area E) and (**b**) the TEM image of the multiphase layer in area F of Figure 7b as well as the corresponding selected area diffraction pattern (SADP).

The interface structure between the 24 h-treated alloy and solidified Al (after immersion for 3 h) was observed using TEM. Figure 8a shows the typical morphology of the intermetallic layer formed after the failure of the oxide film (area E in Figure 7b). In the bright field image (Figure 8a), numerous fine columnar grains (200–500 nm in width, 2–5 µm in length) with heavy strain contrast were observed. These columnar grains were repeatedly analyzed using the SADPs for different grains, and the typical results are shown in the upper right corner of the figure. Using crystal structure analysis, the intermetallic layer was found to be composed of a derivative phase of Co_2_Al_9_. In this research, it was named as (Co, Cr, Mo)_2_Al_9_.

Similarly, the typical structure of the multiphase layer is shown in Figure 8b (area F in Figure 7b). The particles with different morphologies are clearly observed distributed in the Al. SADPs were repeatedly obtained for the particles distributed in different areas of the multiphase layer. Every pair of SADPs that were taken at the same position by tilting the sample can be indexed using a pair of crystallographic directions in Cr_7_Al_45_, indicating that all the particles in the multiphase layer belonged to the same phase. Akin to the phase of (Co, Cr, Mo)_2_Al_9_ in the intermetallic layer, the particles in the multiphase layer were a derivative phase of Cr_7_Al_45_, which was called (Cr, Mo)_7_Al_45_ based on its composition and crystal structure. Therefore, the multiphase layer was composed of Al and (Cr, Mo)_7_Al_45_ phases.

## 4. Discussion

The detailed composition of an oxide film under comparable oxidation conditions has been investigated in previous research [13]. The oxide film in the sample after oxidation for 4 h consists of an outer CoO-rich layer about 200 nm in thickness and an inner Cr_2_O_3_-rich layer about 250 nm in thickness, while in the sample after oxidation for a longer time (longer than 12 h), the oxide film becomes thicker and is gradually transformed into a Cr_2_O_3_-dominated film, which becomes more significant in the alloy with a longer oxidation duration. In contrast, for samples without oxidation, the surfaces are characterized by only small amounts of Cr(OH)_3_ and Co(OH)_2_ and a large fraction of the alloy matrix. 

The results of the corrosion test show that compared to the untreated sample, Co-Cr-Mo alloy has substantially improved corrosion resistance to molten Al through oxidation treatment. This is due to the dense and compact Cr_2_O_3_ oxide film present in the alloy, which is relatively chemically inert to molten Al. In the present study, a 4 h-treated sample exhibited weaker corrosion resistance compared to the sample that underwent a long oxidation treatment. The thinner thickness of the oxide film in the 4 h-treated sample is one of the important reasons for the easier infiltration of molten Al into the matrix. However, it must be noted that there is also a high possibility that a reaction between CoO on the outermost surface of the film and molten Al occurred during immersion. This is supported by the XPS depth profiles of the sample treated for 4 h, in which about 80% of CoO in the film was detected on the outermost surface [11]. In this case, due to the higher reduction activity of CoO by Al compared to Cr_2_O_3_, the following reaction is expected to occur readily once molten Al comes into contact with the oxide film:

3CoO + 2Al → 3Co + Al_2_O_3_ (2)

The film may have been compromised by the reaction between molten Al and CoO, resulting in a much thinner or porous Cr_2_O_3_ film that allows molten Al to easily infiltrate through it. In this case, the sample covered by this film may not be able to withstand prolonged corrosion from molten Al.

It is worth noting that the thickness loss rate of the treated sample in stage III is significantly lower compared to the untreated sample. The reduced thickness loss rate may be attributed to the presence of oxide particles at the cone boundary (Figure 7b). The oxide particles behave in a way that is similar to a porous network covering the reaction products, greatly inhibiting the outwards motion of Cr and Mo from the intermetallic compound. This hypothesis was proven by our EDS measurements of their concentrations in molten Al as shown by Table 1; four locations in Figure 6 for both treated and untreated samples were selected: location A, near layer ② for the untreated sample, location B, inside the cone and near layer ② for the 24 h-treated sample in stage II, location C, near the cone boundary outside the cone for the 24 h-treated sample in stage II, and location D, near layer ② for the 24 h-treated sample in stage III. Compared to the untreated sample, the concentrations of Co, Cr, and Mo in molten Al near layer ② are much higher in the treated sample due to the inhibition of outwards motion of intermetallic particles by the oxide particles around them.

In addition, as shown in Figure 7, due to the absence of oxide particles nearby, the thickness of layer ① (approximately 7 μm) for the untreated sample is much thinner than that of the 24 h-treated treated sample in both stage II (about 60 μm) and stage III (about 40 μm). This is consistent with our supposition that the outwards motion of interfacial reaction products into molten Al was greatly inhibited by the oxide particles, which therefore results in a low dissolution rate or the greater thickness of layer ① due to the higher concentrations of alloy elements near layer ①. 

The inhibiting effect of the oxide particle boundary on the dissolution of intermetallic compounds can also be indirectly seen from the size of the intermetallic phase and the multiple phases. It has been reported that the grain size of both the intermetallic layer and the multiphase layer in the oxidation-treated sample is much larger than that of the bare sample [25]. Such an enlarged particle size for the reactant particle can be reasonably attributed to the inhibited dissolution of its constitute elements. In spite of the significant effect of the oxide film even after its failure, its influence on the thickness loss rate should be much weaker in a flowing molten Al environment, where the cone structure is violated continuously.

## 5. Conclusions

Prior oxidation treatment is effective in enhancing the corrosion resistance of Co-29Cr-6Mo alloy to molten Al due to the coverage of an inert and compact oxide film on the sample surface. 

The protectiveness of the oxide film is better in samples with longer oxidation durations; however, it cannot be further improved with oxidation durations longer than 24 h.

The thickness loss rate of a treated sample even after the full failure of the oxide film is much lower compared to that of untreated sample because the network-aligned oxide particles in the cone boundary greatly inhibit the outwards movements of intermetallic compound particles.

## Figures and Tables

**Table 1:** Atomic composition in different regions in the cross sections of immersed samples.

Location	Co (at.%)	Cr (at.%)	Mo (at.%)	Al (at.%)
A	0.11	0.23	0.05	Balance
B	1.45	0.54	0.37	
C	0.05	0.12	0.07	
D	1.02	0.42	0.15	

## Data Availability

Data available on request from the authors.
